# New dominant *spa* type t2741 in livestock-associated MRSA (CC398-MRSA-V) in Finnish fattening pigs at slaughter

**DOI:** 10.1186/s13756-016-0105-8

**Published:** 2016-03-02

**Authors:** Annamari Heikinheimo, Sophia Johler, Laura Karvonen, Jérôme Julmi, Maria Fredriksson-Ahomaa, Roger Stephan

**Affiliations:** Department of Food Hygiene and Environmental Health, Faculty of Veterinary Medicine, University of Helsinki, Agnes Sjöberginkatu 2, P.O. Box 66, FI 00014 Helsinki, Finland; Institute for Food Safety and Hygiene, Vetsuisse Faculty University of Zurich, Winterthurerstrasse 272, 8057 Zurich, Switzerland

**Keywords:** Livestock-associated MRSA, Pig, Spa type 2741, *fnbB*, *agr*

## Abstract

**Background:**

The emergence of livestock-associated MRSA has become a growing public health concern worldwide. Studies elucidating the population structure, as well as resistance phenotypes and virulence gene profiles of livestock-associated MRSA strains are needed to improve risk assessment and to develop effective control measures. The objective of this study therefore was to determine i) clonal complexes and *spa* types, as well as ii) resistance phenotypes and iii) virulence and resistance gene profiles of livestock-associated MRSA isolated from Finnish fattening pigs at slaughter.

**Methods:**

Fifty MRSA isolates collected from Finnish fattening pigs at slaughter were characterized by *spa* typing and DNA microarray profiling. In addition, antimicrobial susceptibility testing was performed using the Kirby Bauer disk diffusion method.

**Results:**

MRSA isolates were assigned to clonal complexes CC1 (*n* = 4) and CC398 (*n* = 46). One dominant *spa* type (t2741) was present in 33 out of 50 investigated isolates, originating from 15 out of 18 farms. The remaining isolates were assigned to *spa* types t034 (*n* = 7), t108 (*n* = 5), and t011 (*n* = 1). Although each herd exhibited isolates assigned to one clonal complex only, five herds harbored MRSA isolates of either two or three different *spa* types. All tested MRSA isolates were phenotypically resistant to penicillin, oxacillin, cefoxitin, and tetracycline. With the exception of the isolates assigned to t108, all isolates exhibited resistance to clindamycin. On the genomic level, all isolates exhibited *mecA, blaZ/I/R,* and *tetK,* and were assigned to *SCCmec* type V. Many isolates also harbored *tetM* (46/50 isolates), *lnuB* (41/50 isolates), *ermB* (26/50 isolates), and one isolate was positive for *aadD*. DNA microarray profiling showed that all isolates of the dominant CC398/t2741 MRSA-V type belonged to *agr* type I, capsule type 5, and were negative for *fnbB*. Interestingly, one isolate of CC398/t2741 MRSA-V was *agr* negative and also lacked *hld*.

**Conclusions:**

A new dominant LA-MRSA clone (CC398/t2741, *SCCmec* type V) was identified among fattening pigs in Finland. This is the first study identifying t2741 as a common *spa* type in LA-MRSA in pigs.

**Electronic supplementary material:**

The online version of this article (doi:10.1186/s13756-016-0105-8) contains supplementary material, which is available to authorized users.

## Background

Methicillin-resistant *Staphylococcus aureus* (MRSA) is one of the leading causes of nosocomial infections and was first reported in pigs in 2005 [1]. The European Centre for Disease Prevention and Control reported pronounced inter-country variation (0.9–56 %) with regard to the occurrence of MRSA infections in humans in Europe, with Finland exhibiting a very low percentage of invasive MRSA isolates (<5 %) [2]. However, while the proportion of MRSA isolates in all invasive Finnish isolates tested declined from 2011 to 2013, this trend was reversed in 2014 [2].

In the last decade, livestock-associated MRSA (LA-MRSA) have emerged worldwide. LA-MRSA are mostly associated with clonal complex CC398 and can be found at especially high prevalence rates in pigs [3–6]. In Finland, LA-MRSA emerged in 2007, but the proportion of CC398 MRSA in all MRSA isolates detected in humans was very low (0.09 % for 2007–2009) [7]. Data on the prevalence and population structure of Finnish LA-MRSA isolates from pigs is extremely limited. However, seven porcine CC398 isolates obtained in 2008 and 2009 were typed and were exclusively assigned to *spa* type t108 [7].

Epidemiological studies indicate that LA-MRSA also represents an increasing cause of infections in humans [8], LA-MRSA CC398 isolates are likely to have originated in humans as methicillin-susceptible *Staphylococcus aureus*. It has been suggested that while the jump from humans to livestock was followed by acquisition of tetracycline and methicillin resistance, it also led to decreased capacity for human colonization and transmission [9].

MRSA-ST398 isolates were reported to lack important virulence factors such as *pvl*, but were suggested to exhibit enhanced ability to acquire mobile genetic elements [10]. Consistent with this hypothesis, first cases of hospitalizations and death due to infections with MRSA-ST398 isolates exhibiting Panton-Valentine Leukocidin have recently been described [11, 12]. Determining the population structure, as well as resistance phenotypes and virulence gene profiles of LA-MRSA isolates therefore plays a crucial role in risk assessment and provides data essential to the development of effective control measures.

This study aimed to determine i) clonal complexes and *spa* types, as well as ii) resistance phenotypes and iii) virulence and resistance gene profiles of LA-MRSA isolated from Finnish fattening pigs at slaughter.

## Methods

### Sampling, bacterial isolation, and DNA extraction

A total of 29 pig herds from 29 different pig farms from the most dense pig production area of Finland, Western Finland, were included in the study. The farms that the pigs originated from harbored large herds that surpassed average herd size for pig farms in Finland. Some farms obtained the pigs from one seller only, while others obtained pigs from several sellers. From June to July 2015, samples from the anterior nares of pigs at slaughter and from pig carcasses (10cmx10cm surface sampling) were obtained using dry swabbing (Amies Transport Medium, Copan, Brescia, Italy). For each herd, 20 nasal swabs from pigs and 10 swabs from carcasses were taken. Five swabs each were subsequently pooled, resulting in four pooled pig nasal swab samples and two pooled carcass swab samples per herd. Samples were pre-enriched in Mueller-Hinton Broth with 6.5 % NaCl, incubated at 37 °C for 16–24 h. A total of 1 ml was transferred to 9 ml Tryptic soy broth (St. Louis, MO, USA) with cefoxitin (3.5 mg/L) and aztreonam (75 mg/L). After incubation at 37 °C for 16–20 h, a total of 10 μl was transferred on ChromAgar MRSA (Labema, Paris, France) and incubated at 37 °C for 24–48 h. Suspect colonies (one colony from each sample) were confirmed with API Staph (Biomerieux, Paris, France). The antimicrobial susceptibility testing was done by disc diffusion method using cefoxitin discs (EUCAST ecological cut-off values) and PBP2’ test (Oxoid, Hampshire, UK).

A total of 50 MRSA were isolated from nasal swabs of fattening pigs (*n* = 49) and a pig carcass (*n* = 1), originating from 18 different herds/farms. MRSA isolates were grown on 5 % sheep blood agar at 37 °C overnight and chromosomal DNA was extracted using the Qiagen DNeasy Blood and Tissue Kit (Hilden, Germany) in accordance with the manufacturer’s instructions.

### DNA microarray profiling

All presumptive MRSA isolates were further characterized using the Staphytype genotyping 2.0 microarray (Alere, Jena, Germany), which determines the presence or absence of over 300 virulence and resistance genes and their allelic variants. The DNA microarray can also be used as a tool for species confirmation and allows for assignment of isolates to *SCCmec* types and *clonal* complexes [13]. The similarity of the resulting resistance and virulence gene profiles of the different MRSA isolates was visualized using SplitsTree4 (http://www.splitstree.org/) as previously described [14].

### *spa* typing

All isolates were characterized with *spa* typing [15] and *spa* types were assigned using Bionumerics software version 7.5 (Applied Maths, Sint-Martens-Latem, Belgium).

### PCR screening for *lnuB*

Screening for the occurrence of *lnuB* was done by PCR according to Lozano et al. [16].

### Antimicrobial susceptibility testing

Antimicrobial susceptibility testing was performed for eight antimicrobial agents belonging to seven antibiotic classes including anti-staphylococcal beta-lactams (penicillin, oxacillin, cefoxitin), aminoglycosides (gentamicin), macrolides (erythromycin), lincosamides (clindamycin), tetracyclines (tetracycline), fluoroquinolones (ciprofloxacin), and rifamycins (rifampicin) using the Kirby Bauer disk diffusion method. Results were interpreted according to the Clinical and Laboratory Standards Institute [17].

## Results

**Fig. 1:**
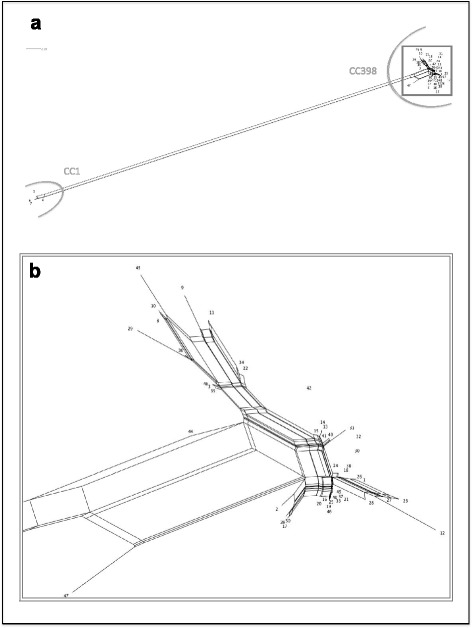
SplitsTree visualizing the similarity of the DNA microarray profiles of the 50 MRSA isolates obtained from fattening pigs in Finland. **a** Overview over the full SplitsTree depicting all 50 isolates. **b** Detail zooming in on the region depicting the 46 highly similar CC398 isolates, while omitting the four CC1 isolates

An overview of the typing and characterization results is provided in Table 1. The 50 MRSA isolates were assigned to clonal complexes CC1 (*n* = 4) and CC398 (*n* = 46), comprising isolates of highly similar resistance and virulence gene profiles (Fig. 1). We identified one dominant *spa* type (t2741) in 33 out of 50 investigated isolates, originating from 15 out of 18 farms. The remaining isolates were assigned to *spa* types t034 (*n* = 7), t108 (*n* = 5), and t011 (*n* = 1). Although each herd harbored only isolates assigned to one clonal complex, we detected either two or three different *spa* in five of the herds. Herd H2 was the only herd harboring MRSA of a clonal complex other than CC398, with all four MRSA isolates assigned to CC1 (*spa* types t127 and t1381).

**Table 1:** Characteristics of 50 *S. aureus* isolates collected from fattening pigs at slaughter in Finland

CC	*spa* typing	SCC*mec*	Herd	Resistance phenotype^a^	Resistance genes^b^	*lnuB*	se^c^	*fnbB*	*agr*	*cap*
CC1 (*n* = 4)	t127 (*n* = 1)	V	H2	PEN, OXA, FOX, TET, CLI, ERY	*mecA, blaZ/I/R, tetK, ermB,*	*-*	*seh*	+	*agrIII*	*cap8*
	t1381 (*n* = 3)	V	H2	PEN, OXA, FOX, TET, CLI, ERY	*mecA, blaZ/I/R, tetK, ermB*	*-*	*seh*	+	*agrIII*	*cap8*
CC398 (*n* = 46)	t011 (*n* = 1)	V	H18	PEN, OXA, FOX, TET, CLI, ERY	*mecA, blaZ/I/R, tetK/M, ermB*	+	-	-	*agrI*	*cap5*
	t034 (*n* = 7)	V	H1, H7, H12, H18	*n* = 6: PEN, OXA, FOX, TET, CLI	*mecA, blaZ/I/R, tetK/M*	+	-	+	*agrI*	*cap5*
			H10	*n* = 1: PEN, OXA, FOX, TET, CLI, CIP	*mecA, blaZ/I/R, tetK/M, aadD*	+	-	+	*agrI*	*cap5*
	t108 (*n* = 5)	V	H3, H16	PEN, OXA, FOX, TET	*mecA, blaZ/I/R, tetK/M*	-	-	+	*agrI*	*cap5*
	t2741 (*n* = 33)	V	H1, H5, H6, H7, H8, H9, H11, H13, H14, H17, H18	*n* = 19: PEN, OXA, FOX, TET, CLI, ERY	*mecA, blaZ/I/R, tetK/M, ermB*	+	-	-	*agrI*	*cap5*
			H1, H4, H10, H15	*n* = 12: PEN, OXA, FOX, TET, CLI	*mecA, blaZ/I/R, tetK/M*	+	-	-	*agrI*	*cap5*
			H9	*n* = 1: PEN, OXA, FOX, TET, CLI, ERY, CIP	*mecA, blaZ/I/R, tetK/M, ermB*	+	-	-	*agrI*	*cap5*
			H17	*n* = 1: PEN, OXA, FOX, TET, CLI, ERY	*mecA, blaZ/I/R, tetK/M, ermB*	+	-	-	*-*	*cap5*

^a^PEN: penicillin, OXA: oxacillin, FOX: cefoxitin, TET: tetracycline, CLI: clindamycin, ERY: erythromycin; CIP: ciprofloxacin

^b^Selected resistance genes detected by DNA microarray (*mecA, blaZ/I/R, ermB, tetK/M, aadD)* or PCR (*lnuB*)

^c^ *se*: genes encoding staphylococcal enterotoxins

All tested MRSA isolates were phenotypically resistant to penicillin, oxacillin, cefoxitin, and tetracycline. With the exception of the isolates assigned to t108, all isolates also exhibited resistance to clindamycin. On the genomic level, all isolates exhibited *mecA, blaZ/I/R,* and *tetK,* and were assigned to *SCCmec* type V (Table 1). Many isolates also harbored *tetM* (46/50 isolates), *lnuB* (41/50 isolates), *ermB* (26/50 isolates), and one isolate was positive for *aadD*.

DNA microarray profiling showed that all isolates of the dominant CC398/t2741 MRSA-V type belonged to *agr* type I, capsule type 5, and were negative for *fnbB* encoding fibronectin-binding protein B (FnBPB). In general, most of the investigated strains lacked several important virulence genes. None of the investigated MRSA isolates carried genes encoding Panton Valentine Leukocidin *(pvl),* toxic shock syndrome toxin (*tst1*), exfoliative toxins (*etA/B/D*), chemotaxis-inhibiting protein (*chp*), staphylococcal complement inhibitor (*scn*), staphylokinase (*sak*), epidermal cell differentiation inhibitors (*edinA/B/C*). In addition, with the exception of the CC1 strains exhibiting *seh,* no enterotoxins genes were detected.

Two major subclones were identified among the isolates of the dominant CC398/t2741 type: The first subclone (*n* = 19, detected in 11 herds) exhibited phenotypic resistance to penicillin, oxacillin, cefoxitin, tetracycline, clindamycin, and erythromycin. In contrast, the second subclone (*n* = 12, detected in 4 herds) lacked erythromycin resistance and the *ermB* gene*.* Only one herd (H1) harbored both subclones. Interestingly, one isolate of CC398/t2741 MRSA-V exhibited genomic and phenotypic traits highly similar to the first subclone, was *agr* negative and also lacked *hld*. A comprehensive compilation of source data, as well as the characterization results obtained by DNA microarray profiling, PCR assays targeting *spa* and *lnuB*, and the antimicrobial susceptibility testing is provided as a Additional files 1 and 2.

## Discussion

Isolates of CC398/t2741 exhibiting *SCCmec* type V were dominant among the investigated MRSA isolates. To date, few individual CC398/t2741 MRSA-V isolates have been reported in association with human hosts [8, 18, 19]. The National Institute of Health and Welfare reported in its infection news that while 72 % of the recent CC398-associated infections in humans in Finland were caused by strains of *spa* type t034, 7 % of the infections were associated with t2741 strains (https://www.thl.fi/en/web/thlfi-en). However, this is the first study identifying t2741 as a common *spa* type in LA-MRSA in pigs. In addition, no *fnbB*-negative or *agr/hld*-negative t2741 isolates have been previously reported.

Previous studies suggest that MRSA-CC398 vary only very little in core genome-encoded surface and secreted genes, with *fnbB* being absent in only 1 out of 76 analyzed isolates [20]. FnBPB belongs to the microbial surface components recognizing adhesive matrix molecules (MSCRAMMs). It enables *S. aureus* to adhere to components of host cells and plays a role in invasion [21]. The combined function of FnBPA and FnBPB was shown to be essential for the induction of severe infection [22]. The accessory gene regulator (*agr*) represents a quorum sensing system mediating the staphylococcal virulon. While dysfunction of the *agr* system was suggested to promote proliferation and survival of *S. aureus* within the infected host, it may be counter-adaptive outside of infected host tissues and may decrease the chances of long-term survival [23].

The other *spa* types detected in this study (t011, t034, t108) are commonly found in LA-MRSA isolated from pigs in Europe [3, 19, 24–26]. Interestingly, t108 was shown to be transmissible between pigs and pig farmers, as well as between humans [1]. There are also indications that *spa* type t108 might have been the predominant *spa* type in LA-MRSA strains isolated from Finnish pigs, when CC398 first emerged in Finland [7].

Nineteen of the MRSA CC398 isolates (t034, t2741) in this study exhibited the unusual resistance phenotype L^R^/M^S^ (lincosamide resistance, macrolide susceptibility). These isolates were exclusively associated with *spa* types t034 (7/7 isolates) and t2741 (12/33 isolates). Recently, this unusual resistance phenotype has increasingly been observed among strains from animal origin [27]. In MRSA CC398 isolates lacking *erm* genes, this resistance phenotype has been suggested to be conferred by *lnuA/B/C/D/G* [16]. This is consistent with the findings in this study, showing that the L^R^/M^S^ phenotype was associated with isolates lacking *ermB*, but harboring *lnuB.*

The fact that only very few enterotoxin genes and no *pvl*, *etA/B/D,* and *tst-1* genes were detected in this study is consistent with previous studies indicating that *S. aureus* of ST398 lack certain virulence factors, which may account for these isolates being only rarely associated with invasive disease in humans (Schijffelen et al., 2010). However, MRSA ST398 isolates were suggested to exhibit an enhanced ability to acquire mobile genetic elements and may therefore rapidly acquire virulence determinants increasing the virulence of these strains in the human host [10]. As first cases of severe illness and death due to infections with MRSA-ST398 isolates exhibiting Panton-Valentine Leukocidin have been described [11, 12], the occurrence of MRSA ST398 isolates in livestock and human hosts and associated virulence and resistance gene patterns needs to be closely monitored.

## Conclusions

A new dominant LA-MRSA clone (CC398/t2741, *SCCmec* type V) was identified among fattening pigs in Finland. This is the first study identifying t2741 as a common *spa* type in LA-MRSA in pigs. As strains of ST398 were reported to easily acquire virulence determinants, monitoring of virulence and resistance profiles of LA-MRSA in livestock and human carriers in close contact to livestock production is crucial to recognize emerging hypervirulent clones and to develop comprehensive and efficient control strategies.

## Statement on ethics

There is no consideration about the sampling of carcasses because animal rights do not apply to slaughtered farm animals. Nasal swabs were taken after stunning and therefore comply with national guidelines.

## Additional files

Additional file 1: Overview on phenotypic and genotypic characteristics of each strain. (XLSX 31 kb) Additional file 2: Complete microarray data for each strain. (XLSX 86 kb)
